# Progress in Gynæcology

**Published:** 1899-08-26

**Authors:** 


### Progress in Gynæcology.

(*Continued from page* 351.)

**Hysterectomy as a Conservative Operation.**—Bland Sutton,[11] writing on this subject, says that the health of a woman during the sexual period of life suffers less from the loss of her uterus than after complete removal of the ovaries; and that this fact is producing extraordinary modifications in the treatment of diseases of the uterus, Fallopian tubes, and ovaries. He points out that the uterus is a hollow muscle of the unstriped variety, and lined with a mucous membrane having peculiar characters and known as the endometrium. The uterus also resembles the other hollow muscles, as the heart, bladder, &c., in that it acts independently of the will, and undergoes more or less rhythmic changes commonly known as dilatation and contraction. The duration of the cycle differs in all, and in the uterus it occupies three-fourths of a year with a long interval for rest. The uterus is singular amongst hollow muscles, not only in the excessive length of its cycle or beat, but also in the fact that its epithelium is shed with great regularity once a month, save when it is occupied by its long diastole, that is during pregnancy. He then points out that we have positive knowledge that the menstrual organ is the endometrium, and that menstruation is governed in some obscure way by the ovary. The ovarian influence is not confined to the control of menstruation, but has some effect on what is known as the vaso-motor functions, so that when the ovarian control is suddenly removed during the sexual period of life those singular disturbances, "the flushes," annoy the patient. That this flushing is not due simply to arrest of menstruation is absolutely demonstrated by the fact that complete extirpation of the uterus arrests menstruation as thoroughly as complete removal of both ovaries, yet "conservative hysterectomy"—that is removal of the uterus with preservation of at least one ovary—is not followed by the disagreeable vaso-motor disturbances. Bland Sutton recommends extirpation of the uterus under the following circumstances: (1) In Hæmatometra, due to congenital atresia of some part of the cervical canal. Such a uterus, he says, is not only useless for procreation, but it is a source of anxiety, much suffering, and a grave danger. Its extirpation at the outset is a conservative act. (2) For fibrosis of the uterus. The subjects of this condition are usually mothers between 35 and 45 years of age, complaining of menorrhagia, which in some cases lasts from 14 to 18 days, and at times is so profuse as to nearly bleed them to death. When the uterine canal is dilated artificially the cervix tears rather than stretches, and the endometrium is quite smooth, but the walls of the uterus are hard and resisting. In three cases of this kind, in which long-continued drug treatment, varied with annual and bi-annual curettings had failed, the uterus was removed by the vaginal route with success and permanent good consequences. In these three cases the uterus was larger than normal, with thick, tough walls. On section the arteries stand out prominently exposing their thickened coats; microscopically it is seen that the muscle tissue is replaced by an abnormal growth of fibrous tissue. C. E. Purslon[12] reports two cases of intractable uterine hæmorrhage for which vaginal hysterectomy was performed, one of which was examined microscopically and the changes above described were well marked. (3) For uterine fibro-myomata: After reviewing the different methods that have been adopted in the treatment of these tumours Bland Sutton regards removal of the uterus and preservation of the ovaries as the best treatment, and emphasises the importance of bearing in mind (when advising operative interference in these cases) the great influence of the ovaries. He concludes by saying that a careful consideration of all the facts makes it clear that the uterus can only be considered as a receptacle or reservoir wherein oösperms may develop; it is secondary and certainly subservient to the ovaries. It is not a vital organ, and its removal entails two physiologic sequelæ in women during the sexual period of life, namely, amenorrhœa and sterility.

**The Treatment of Uterine Hæmorrhage.**—H. J. Boldt[3] has been observing the effect of stypticin in the treatment of uterine hæmorrhage. The drug is obtained from narcotine by oxidation with manganese dioxide and sulphuric acid, and is closely allied chemically to hydrastinine; it occurs as a yellow, odourless powder of an intensely bitter taste, and readily soluble in water. From observations made in 70 cases he draws the following conclusions: That uterine hæmorrhages resulting from no demonstrable pathologic anatomical condition, whether they occur in virgins or during the climacteric period, are favourably influenced, and so are hæmorrhages due to changes in the uterine mucosa that persist after curetting has been performed. On the other hand, in cases where no scraping has been done, the remedy is of little or no use. A satisfactory action may also be expected in hæmorrhage which, in consequence of inflammatory conditions in the pelvis, occur without any special pathological changes in the endometrium. On the other hand, hæmorrhages traceable to neoplasms within or around the uterus are not at all or but very little influenced. Abram Brothers[14] recommend intrauterine vapourisation for the treatment of uterine hæmorrhage. After dilatation of the cervix, a uterine catheter properly protected is introduced to a point within a short distance of the fundus uteri, through which super-heated steam (212-230 Fahr.) is brought into direct contact with the uterine interior. Brothers has used it in seventy-eight cases, obtaining successful results in sixty-four. As a hæmostatic it has been employed most successfuly in cases of non-malignant post-climacteric uterine hæmorrhage. It has proved curative in the various irregular bleedings met with in connection with catarrhal fungoid or hæmorrhagic endometritis. It acts as a palliative measure in certain cases of fibroid tumour or inoperable carcinoma asso ciated with hæmorrhage. Pelk[15] recommends the use of thyroid extract in the treatment of uterine hæmorrhage associated with fibroids, and reports ten cases, in nine of which improvement followed its use.

**Malignant Disease of the Uterus.**—Jessett,[16] writing on this subject, urges the absolute necessity of making a thorough vaginal examination in all cases of women complaining of menorrhagia, purulent discharge, or dysmenorrhœa, so that early malignant disease may be detected in that stage in which it can be operated upon with success. He advocates complete vaginal hysterectomy for these cases in preference to the supravaginal operation, as after the latter, even if the diseased portion is removed, patients are liable to stenosis of the uterine canal, and suffer intensely from dysmenorrhœa, and the mortality after this operation is quite as great as after the major one of vaginal hysterectomy. As to the prognosis after operation, much depends upon the position of the disease, the extent of it, and the length of time it has existed. Cases of malignant disease of the os and lower part of the cervix are undoubtedly the most suitable for operation, and in these cases the prognosis may be looked upon as favourable. The next most favourable position for the disease to be situated for successful operative interference is the body of the uterus. The disease may arise either as a primary growth starting from the mucous glands, or from a polyp taking a malignant action, or from a submucous fibroid becoming malignant; the last form Jessett believes is far more common than is usually supposed. The most unfavourable cases for operation are those in which the disease commences in the cervical canal, the reason of this is that the tissues in the roof of the vagina, and the lymphatics therein become very quickly infected and the uterus, in consequence, becomes fixed early. So long, however, as the uterus is freely moveable, even if the mucous membrane of the fornices is implicated, Jessett advises operation. He recommends total extirpation of the uterus in all cases in preference to supravaginal amputation of the cervix. As to the more radical operation, such as that suggested by Halstead, of removing the diseased uterus by the abdomen, and dissecting out all the obturator, sacral, and lumbar glands, he thinks that at present the mortality after such an operation would probably more than counterbalance the benefit to those that are saved. Dr. Halliday Croom[17] is averse to operative measures in uterine cancer unless the case is seen at a very early stage; and says that he thinks, judging from his experience at Edinburgh, that the surgical method of dealing with uterine cancer has done little to ameliorate suffering or prolong life.

11 Brit. Med. Jour. April 8, 1899. 12 Brit. Med. Jour, June 10, 1899. 13 Merck's Archives, Feb., 1899. 14 New York Med. Jour., May, 1899. 15 New York Med. News, Jan. 14, 1899. 16 Med. Chron., May, 1899. 17 Med. Chron., Mar., 1899.

